# Supplementary material for: Actomyosin polarisation through PLC-PKC triggers symmetry breaking of the mouse embryo
Source: Nat Commun. 2017 Oct 13;8:921. doi: 10.1038/s41467-017-00977-8 (PMC5640629; doi:10.1038/s41467-017-00977-8)
Supplement: Supplementary file 1 — Supplementary Information [file 41467_2017_977_MOESM1_ESM.pdf]

Supplementary table 1. sequence of primers used in this study to clone the different mutant constructs

Primer	Sequence (5' to 3')
GFP-Myl12b-T18DS19D, Forward	GCGCAGATGACAATGTGTTCGCCATGTTTGACCAGTCCC
GFP-Myl12b-T18DS19D, Reverse	CACATTGTCATCTGCGCGCTGAGGGCGCTTCTTGGTG
RhoA-Q63L, Forward	ACGTGAATTCATGGAGCAGAAGCTGATCTC
RhoA-Q63L, Reverse	ACGTACTAGTTCACAAGACAAGGCACCCAG
CIB1, Forward	ACGTGAATTCATGAATGGAGCTATAGGAGGTGACC
CIB1, Reverse	ACTTGGATCCAGCTTGAGCTCGAGATCTGAGTC
Kras-CAAX motif, Forward	ACGTCTCGAGGAAAGATGAGCAAAGATGGTAAAAAG
Kras-CAAX motif, Reverse	ACGTGGATCCCCAAATACTCATTCTGATGTTTTAAATG
CRY2-mCherry, Forward	ACGTGAATTCATGAAGATGGACAAAAAGACCATCGTCTG
CRY2-mCherry Reverse	ACGTGGATCCAGCTTGAGCTCGAGATCTGAGTC
PKC-Kinase domain, Forward	ACGTGGATCCACAGACTTCAACTTCCTCATGGTG
PKC-Kinase domain, Reverse	AGCAGATACGAATGGCTACATTTTGG
Prkca wild-type, Forward	GGACGAGAATTCACCATGGCTGACGTTTACCCGGC
Prkca wild-type, Forward	TTTTAAGCGGCCGCTCATACTGCACTTTGCAAGA
Prkca-A25E A	GGCAGATCTGAATTCACCATGGC
Prkca-A25E B	GCCTCAGCTCCCCTTTGCGG
Prkca-A25E C	CCGCAAAGGGGAGCTGAGGC
Prkca-A25E D	TCCGCGGCCGCTCATACTGC
CRY2-mCherry-RhoA-L63-C190R, C190R mutagenesis Forward	GAAGAAAAAATCTGGGCGCCTTGTCTTGTG AACTAGTTCTAGAGCGG
CRY2-mCherry-RhoA-L63-C190R, C190R mutagenesis Reverse:	TTCACAAGACAAGGCGCCCAGATTTTTTCTT CCCACGTCTAGCTTGC
CRY2-mCherry-RhoA-L63-C190R,Forward:	ACGTGAATTCCATGGAGCAGAAGCTGATCT C
CRY2-mCherry-RhoA-L63-C190R, Reverse:	ACGTACTAGTTCACAAGACAAGGCACC
LifeAct-eGFP forward:	CTTCGAATTCACGATGGGCGTGG
LifeAct-eGFP	TGGATCCGGCTCCTCCTTGC

Reverse:	
Ezrin-Ruby Forward:	TTCGAATTCGCCACCATGCCCA
Ezrin-Ruby Reverse:	TGCAGAATTCCCATGGCCTCGA

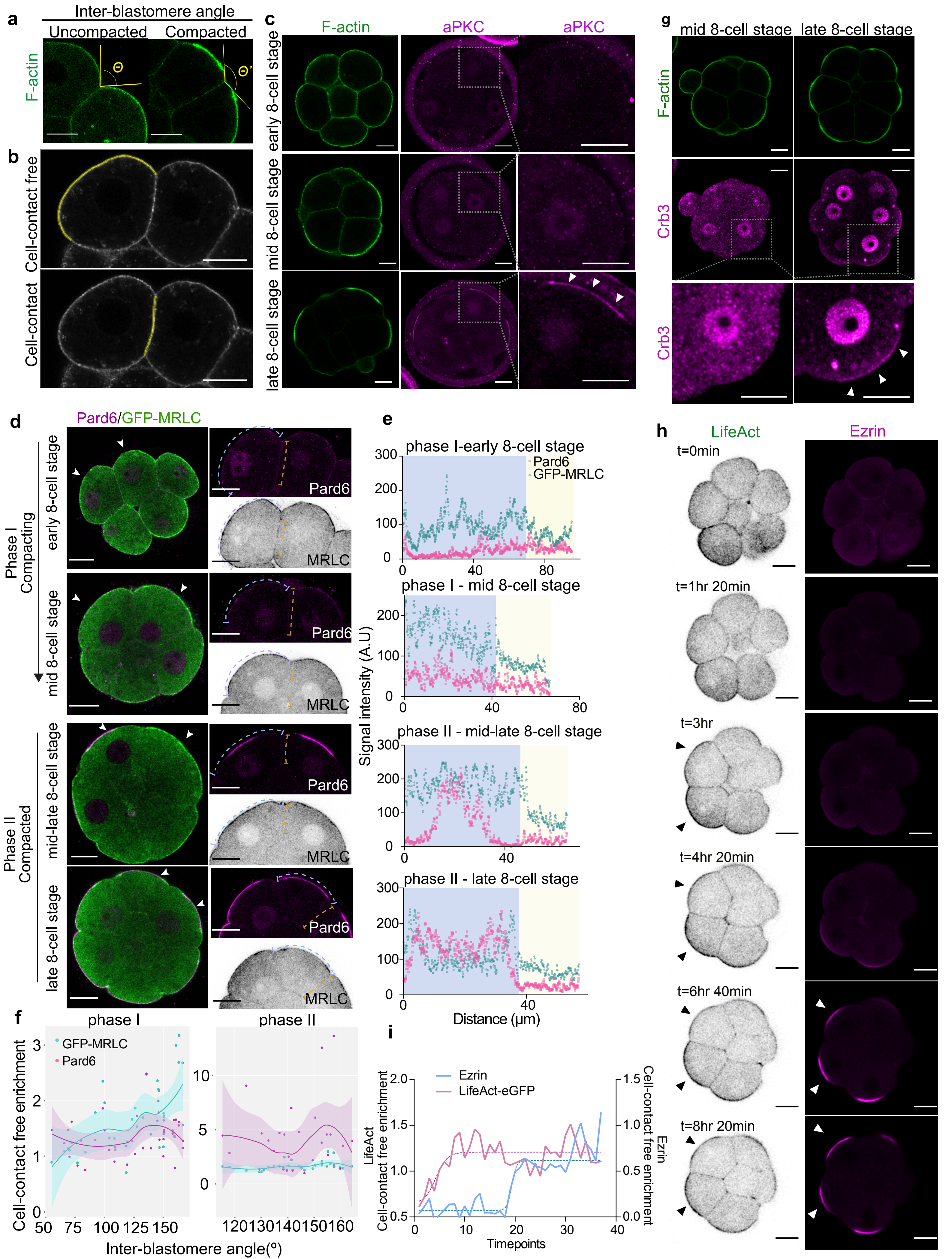
a
Inter-blastomere angle
Uncompacted
Compacted
F-actin
Θ
Θ'
b
Cell-contact free
Cell-contact
c
F-actin
aPKC
aPKC
early 8-cell stage
mid 8-cell stage
late 8-cell stage
g
mid 8-cell stage
late 8-cell stage
F-actin
Crb3
Crb3
d
Pard6/GFP-MRLC
Phase I
Compacting
early 8-cell stage
mid 8-cell stage
Phase II
Compacted
mid-late 8-cell stage
late 8-cell stage
Pard6
MRLC
e
phase I-early 8-cell stage
Pard6
GFP-MRLC
phase I - mid 8-cell stage
phase II - mid-late 8-cell stage
phase II - late 8-cell stage
Signal intensity (A.U)
Distance (μm)
h
LifeAct
Ezrin
t=0min
t=1hr 20min
t=3hr
t=4hr 20min
t=6hr 40min
t=8hr 20min
f
phase I
phase II
GFP-MRLC
Pard6
Cell-contact free enrichment
Inter-blastomere angle(°)
i
Ezrin
LifeAct-eGFP
LifeAct
Cell-contact free enrichment
Ezrin
Cell-contact free enrichment
Timepoints

Supplementary Figure 1. Actomyosin apical asymmetry precedes apical domain maturation.

(**a**) Examples of an uncompacted and compacted 8-cell stage mouse embryo showing the IEA. (**b**) Examples of the ROIs used to plot circumferential line profiles of F-actin, GFP-MRLC and Pard6.A line of 0.8μm width was drawn to cover the whole cortex. (**c**) Localisation pattern of F-actin and PKCζ during 8-cell stage development. Squares indicate the magnified region. Arrows indicate the apically localised PKCζ. (**d**) Embryos expressing GFP-MRLC were fixed at the early, mid and late 8-cell stage and immunostained for Pard6. Arrowheads indicate the magnified blastomeres. Yellow dotted lines indicate cell-cell contact regions and blue dotted lines indicate cell-contact free surface. (**e**) Circumferential line profiles of GFP-MRLC and Pard6 fluorescence intensity in embryos from panel d. Yellow indicates cell-cell contact regions and blue cell-contact free domain. (**f**) Cell-contact free enrichment of GFP-MRLC and Pard6 plotted against the IEA. (N=14 embryos, 4 independent experiments). LOWESS plot were used to display the tendency of different datasets. Shadow indicates the 95% confidence interval. (**g**) Localisation pattern of F-actin and Crb3 during 8-cell stage development. Squares indicate the magnified region. Arrows indicate the apically localised Crb3 (N=8 embryos, 2 independent experiment). (**h**) Snapshots of time-lapse recordings of embryos expressing Ezrin-Ruby and LifeAct-eGFP. Arrows indicate the polarised localisation of LifeAct-eGFP or Ezrin-Ruby. (**i**) Quantification of Ezrin and LifeAct-eGFP cell-contact free enrichment in embryos from panel h (N=10 embryos, 3 independent experiments). Quantifications were fitted into a "Boltzmann sigmoidal" model based curve (R^2= 0.8886 for Ezrin-Ruby, R^2=0.5198 for LifeAct-eGFP). All scale bars, 15μm

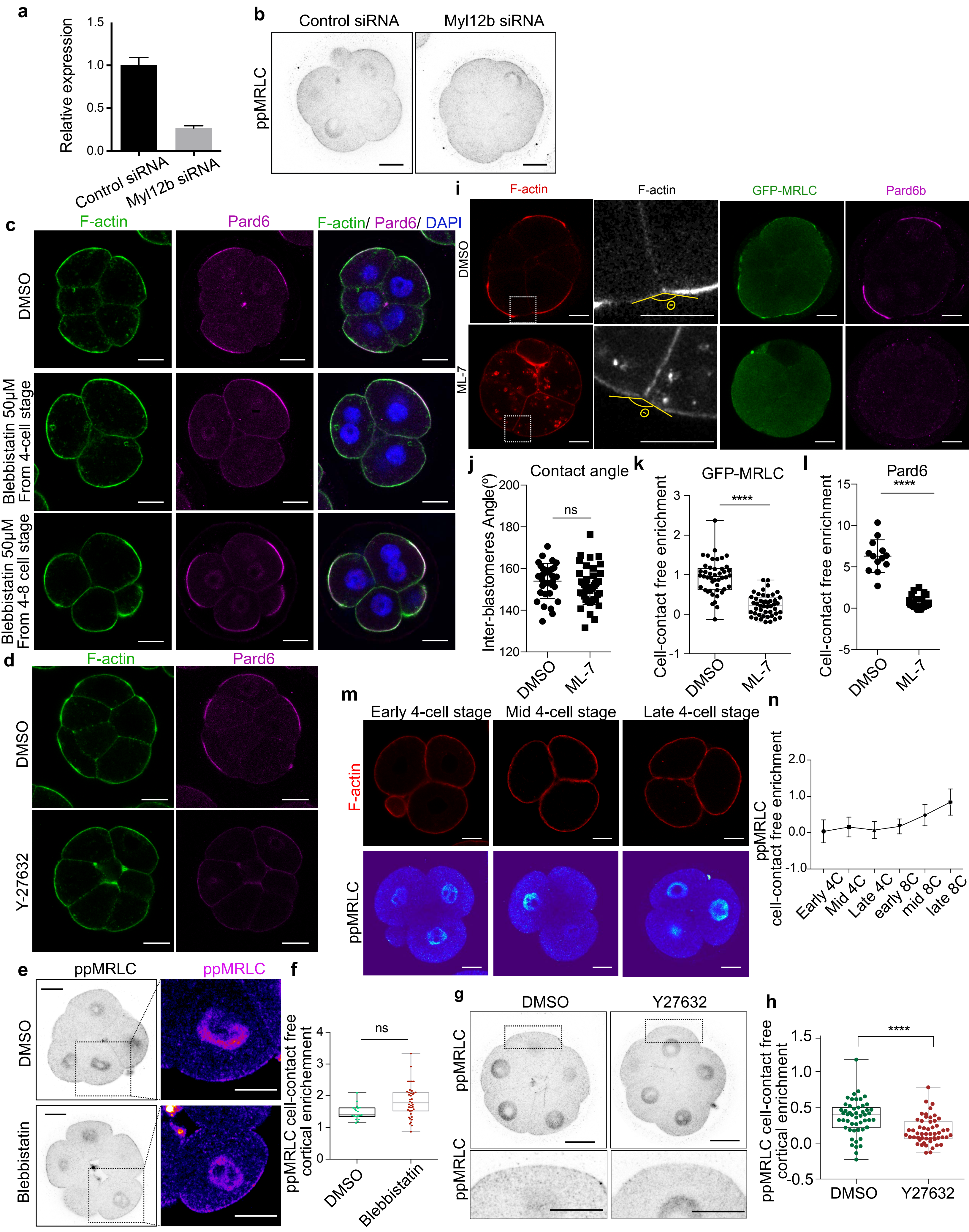
a
Relative expression
Control siRNA
Myl12b siRNA
b
Control siRNA
Myl12b siRNA
ppMRLC
c
F-actin
Pard6
F-actin/ Pard6/ DAPI
DMSO
Blebbistatin 50µM From 4-cell stage
Blebbistatin 50µM From 4-8 cell stage
d
F-actin
Pard6
DMSO
Y-27632
e
ppMRLC
ppMRLC
DMSO
Blebbistatin
f
ppMRLC cell-contact free cortical enrichemnent
ns
DMSO
Blebbistatin
g
DMSO
Y27632
ppMRLC
ppMRLC
h
ppMRLC cell-contact free cortical enrichment
****
DMSO
Y27632
i
F-actin
F-actin
GFP-MRLC
Pard6b
DMSO
ML-7
j
Contact angle
Inter-blastomeres Angle(º)
ns
DMSO
ML-7
k
GFP-MRLC
Cell-contact free enrichment
****
DMSO
ML-7
l
Pard6
Cell-contact free enrichment
****
DMSO
ML-7
m
Early 4-cell stage
Mid 4-cell stage
Late 4-cell stage
F-actin
ppMRLC
n
ppMRLC cell-contact free enrichment
Early 4C
Mid 4C
Late 4C
early 8C
mid 8C
late 8C

Supplementary Figure 2. Apical actomyosin organisation is required for Par complex recruitment.

(**a**) Verification of Myl12b siRNA knockdown efficiency by qPCR. (**b**) Control siRNA injected embryos and Myl12b siRNA injected embryos were fixed at the late 8-cell stage and immunostainned for ppMRLC. (**c**) DMSO (control) and Blebbistatin-treated embryos fixed at the late 8-cell stage and immunostained F-actin and Pard6 (N=8 embryos for DMSO and N=9 embryos for Blebbistatin, 2 independent experiments). (**d**) DMSO (control) and Y-27632-treated embryos at the 4-8 cell stage were fixed at the late 8-cell stage and immnostained F-actin and Pard6 (N=18 embryos for DMSO and N=20 embryos for Y-27632, 3 independent experiments). (**e**) Early 8-cell stage embryos were treated with DMSO (control) or Blebbistatin, fixed at the late 8-cell stage and immunostained for ppMRLC. Squares indicate the magnified region. (**f**) Quantification of cell-contact free cortical ppMRLC enrichment in embryos from panel e. ns=not significantly different (N=10 embryos for DMSO and N=12 embryos for Blebbistatin, 2 independent experiments). (**g**) DMSO (control) and Y-27632 treated embryos were fixed at the late 8-cell stage and immunostained for ppMRLC. (**h**) Quantification of cell-contact free cortical enrichment of ppMRLC in embryos from panel g. ****$p<0.0001$; Mann-Whitney test. (N=27 embryos for DMSO and N=30 embryos for Y-27632, 2 independent experiments). (**i**) 2-cell stage embryos were injected with GFP-MRLC, treated with DMSO or ML-7 at the mid 8-cell stage, fixed at the late 8-cell stage and immunostained for F-actin, GFP-MRLC and Pard6. Squares indicate the magnified region. (**j-l**) Quantification of the IEA (j), GFP-MRLC cell-contact free enrichment (k), and Pard6 cell-contact free enrichment (l) in embryos from panel i. ns=not significantly different, ****$p<0.0001$, unpaired student's t-test. (N=9 embryos for DMSO and N=10 embryos for ML-7, 4 independent experiments). (**m**) Early (0-1hr post-cell division), mid (3-4 hr post-cell division) and late (9-10 hr post-cell division) 4-cell stage embryos fixed and immunostained F-actin and ppMRLC. (**n**) Quantification of ppMRLC cell-contact free enrichment in embryos from panel m. (N=8-10 embryos, 2 independent experiments). Error bars represent s.d. All scale bars, 15μm.

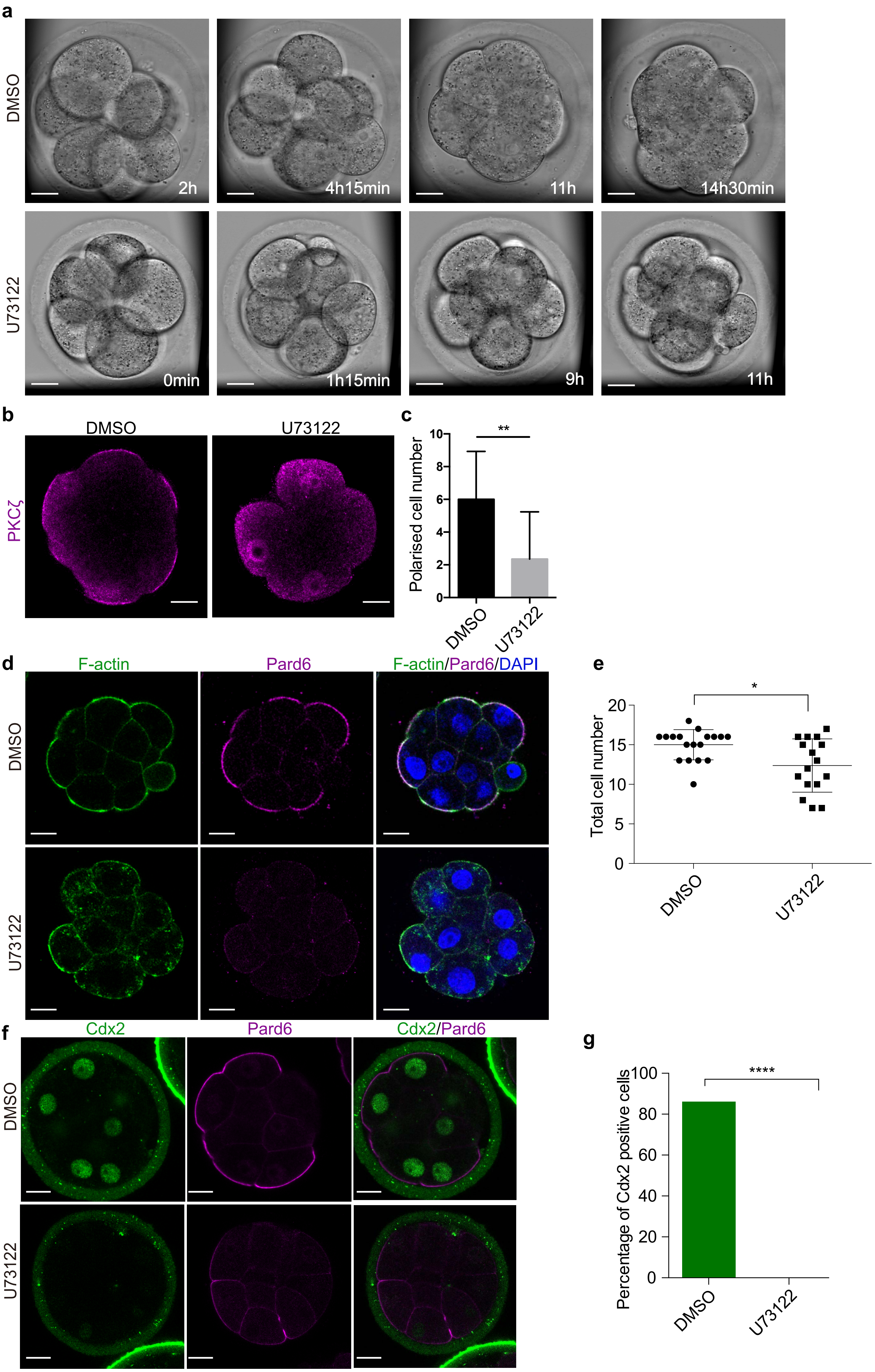

a
DMSO
2h
4h15min
11h
14h30min
U73122
0min
1h15min
9h
11h
b
DMSO
U73122
PKCζ
c
**
Polarised cell number
0
2
4
6
8
10
DMSO
U73122
d
F-actin
Pard6
F-actin/Pard6/DAPI
DMSO
U73122
e
*
Total cell number
0
5
10
15
20
DMSO
U73122
f
Cdx2
Pard6
Cdx2/Pard6
DMSO
U73122
g
****
Percentage of Cdx2 positive cells
0
20
40
60
80
100
DMSO
U73122

Supplementary Figure 3. PLC inhibition abolishes compaction and polarisation.
(**a**) Snapshots of time-lapse recordings of embryos treated with DMSO and U73122 (N=72 embryos for DMSO and N=127 embryos for U73122, 12 independent experiments). (**b**) Embryos treated with DMSO or U73122 were immunostained for PKCζ. (**c**) Quantification of PKCζ positive cell numbers in embryos from panel b. *$p<0.05$, Mann- Whitney test. (N=11 embryos for DMSO and N=28 embryos for U73122, 2 independent experiments). (**d**) Embryos treated with DMSO or U73122 immunostained for F-actin and Pard6 at the 16-cell stage. (**e**) Total cell numbers in embryos from panel d. *$p<0.05$, ns = not significantly different, two-tailed unpaired Student's t-test. (N=18 embryos for DMSO and N=16 embryos for U73122, 3 independent experiments). (**f**) DMSO and U73122 overexpressing embryos fixed at the 16-cell stage and immunostained for Cdx2 and Pard6. (**g**) Percentage of Cdx2 positive cells in embryos from panel f (N=10 embryos for each group). Error bars represent s.d. All scale bars, 15μm.

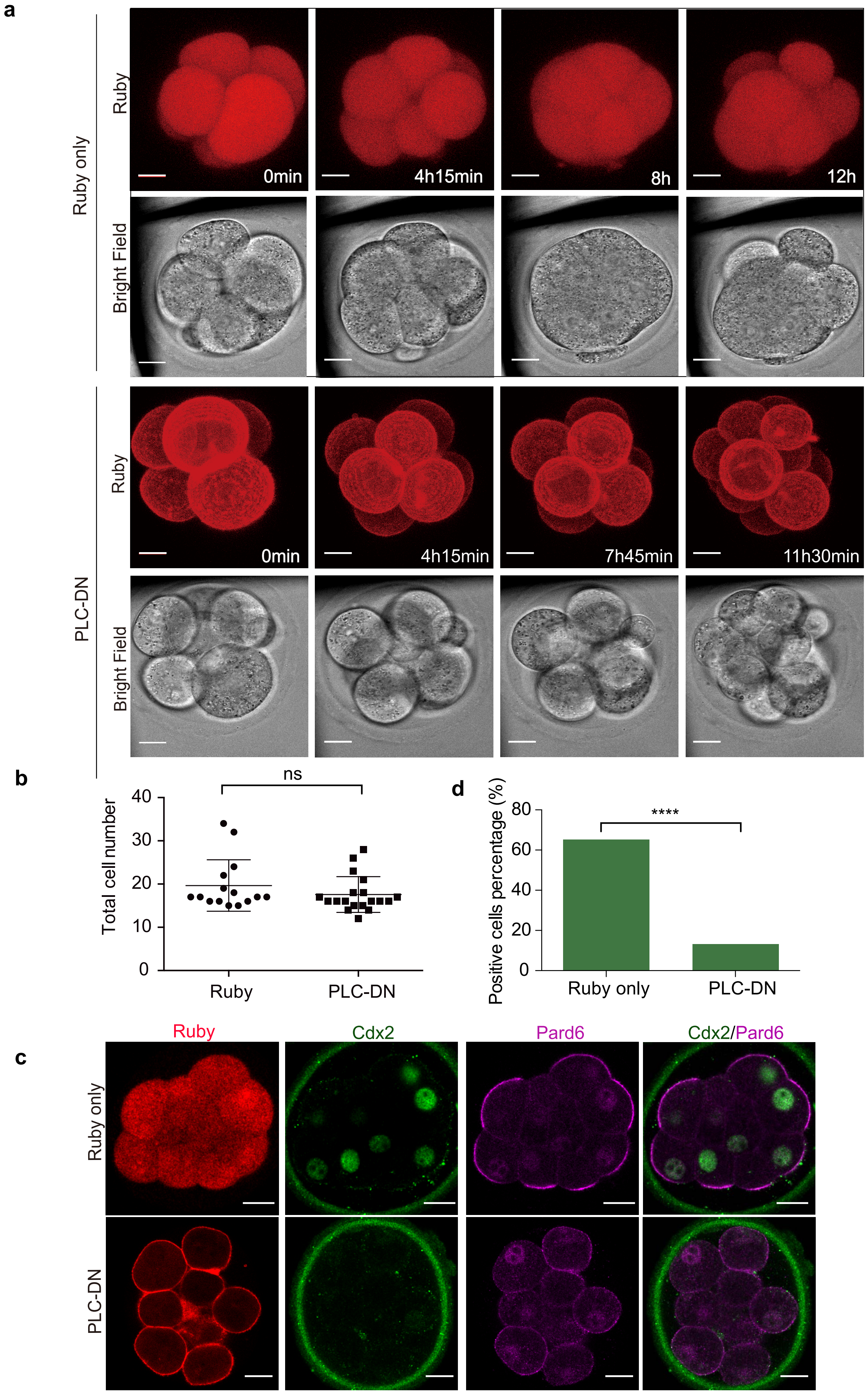
a
Ruby only
Ruby
Bright Field
0min
4h15min
8h
12h
PLC-DN
Ruby
Bright Field
0min
4h15min
7h45min
11h30min
b
ns
Total cell number
40
30
20
10
0
Ruby
PLC-DN
d
Positive cells percentage (%)
80
60
40
20
0
****
Ruby only
PLC-DN
c
Ruby
Cdx2
Pard6
Cdx2/Pard6
Ruby only
PLC-DN

Supplementary Figure 4. Expression of a PLC dominant negative inhibits compaction and polarisation.

(**a**) Snapshots of time-lapse recordings of embryos overexpressing Ruby or PLC-DN (**b**) Total cell numbers in embryos from panel a. Each dot represents an embryo. ns = not significantly different, Mann-Whitney test. (N=15 embryos for Ruby and N=19 embryos for PLC-DN, 4 independent experiments). (**c**) Ruby and PLC-DN overexpressing embryos fixed at the 16-cell stage and immunostained for Cdx2 and Pard6 (**d**) Percentage of Cdx2 positive cells in embryos from panel c. (N=8 embryos for each group, 4 independent experiments). All scale bars, 15μm.

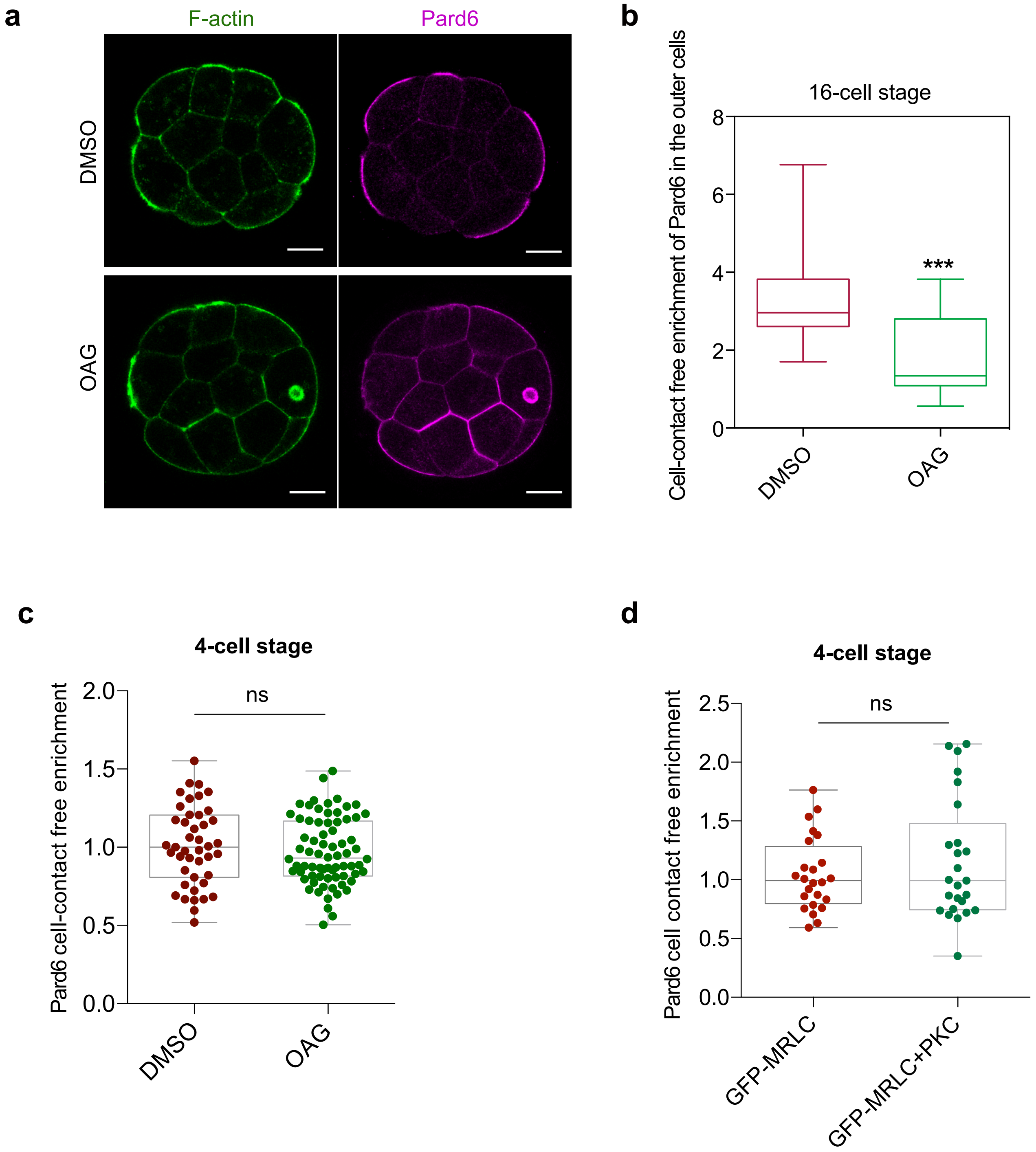

a
F-actin
Pard6
DMSO
OAG
b
16-cell stage
Cell-contact free enrichment of Pard6 in the outer cells
0
2
4
6
8
***
DMSO
OAG
c
4-cell stage
Pard6 cell-contact free enrichment
0.0
0.5
1.0
1.5
2.0
ns
DMSO
OAG
d
4-cell stage
Pard6 cell contact free enrichment
0.0
0.5
1.0
1.5
2.0
2.5
ns
GFP-MRLC
GFP-MRLC+PKC

Supplementary Figure 5. PKC ectopic activation triggers expansion of the Pard6 domain. (**a**) Embryos treated with DMSO or OAG were fixed at the 16-cell stage and immunostained for F-actin and Pard6. (**b**) Quantification of the cell-contact free enrichment of Pard6 in embryos from panel a. ***$p<0.001$, Mann-Whitney test. (N=29 measurements and 6 embryos (DMSO group) and N=17 measurements and 4 embryos (OAG group), 3 independent experiments). (**c**) Quantification of the cell-contact free enrichment of Pard6 in embryos from Fig. 5c. ns=not significantly different, two-tailed unpaired Student's t-test. (N=12 embryos for DMSO and N=26 embryos for OAG, 4 independent experiments). (**d**) Cell-contact free enrichment of Pard6 in embryos from Fig. 5f. Each dot represents an individual blastomere. ns = not significantly different, Mann-Whitney test. (N=18 embryos for GFP-MRLC, N=19 embryos for GFP-MRLC+PKCα-A25E, 3 independent experiments). For all panels data is shown as individual data points with Box and Whiskers graph (bottom: 25%; top: 75%; line: median; whiskers: min to max). All scale bars, 15µm.

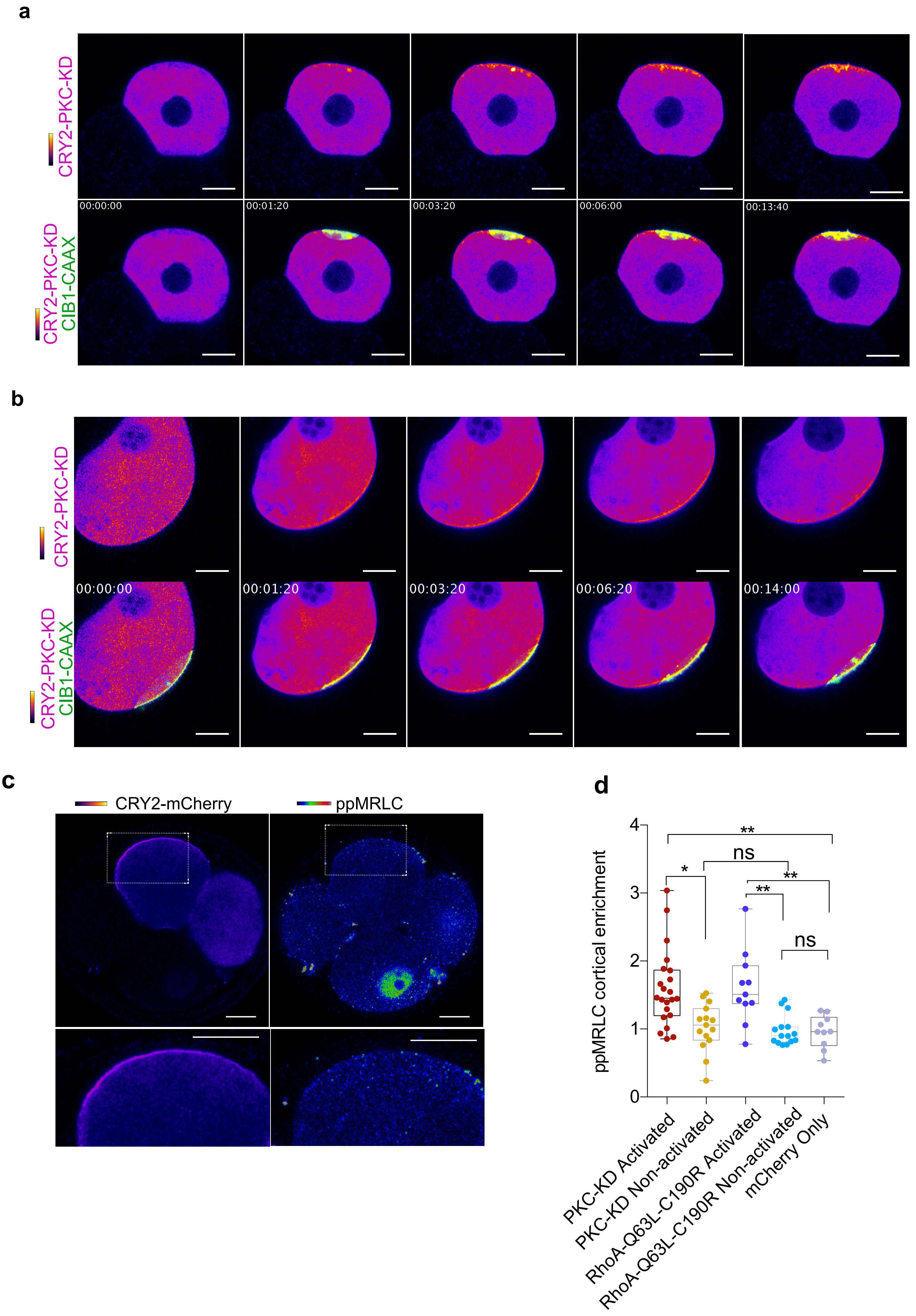
a
CRY2-PKC-KD
CRY2-PKC-KD
CIB1-CAAX
00:00:00
00:01:20
00:03:20
00:06:00
00:13:40
b
CRY2-PKC-KD
CRY2-PKC-KD
CIB1-CAAX
00:00:00
00:01:20
00:03:20
00:06:20
00:14:00
c
CRY2-mCherry
ppMRLC
d
ppMRLC cortical enrichment
0
1
2
3
4
**
ns
*
**
**
ns
PKC-KD Activated
PKC-KD Non-activated
RhoA-Q63L-C190R Activated
RhoA-Q63L-C190R Non-activated
mCherry Only

Supplementary Figure 6. PKC activation regionally activates the actomyosin meshwork. (**a**) Snapshots of CRY2-mCherry-PKC-KD activation (embryo displayed in Fig. 5k-F-actin). (**b**) Snapshots of CRY2-mCherry-PKC-KD activation (embryo displayed in Figure 5k-ppMRLC). (**c**) Embryos expressing CIB1-Zsgreen-CAAX and CRY2-mCherry and illuminated with blue light (using the same conditions as for CRY2-mCherry-PKC-KD) were immunostained for ppMRLC. (**d**) Quantification of cortical ppMRLC level in the indicated conditions. For activating conditions, ppMRLC level was quantified in the regions that showed membrane CRY2 translocation. In non-activating conditions, ppMRLC was quantified in the cell-cell contact free domain. Signal intensity was normalised to the ppMRLC levels in the entire blastomere. *$p<0.05$, **$p<0.01$, ns=not significantly different; Mann-Whitney test. (N=17 embryos for PKC-KD activated region, N=9 embryos for PKC-KD non-activated region, N=7 embryos for RhoA-Q63L-C190R activated region, N=13 embryos for RhoA-Q63L-C190R non-activated region and N=10 embryos for mCherry only, 3 independent experiments for mCherry only, 2 independent experiments for all the other conditions). All scale bars, 15μm.

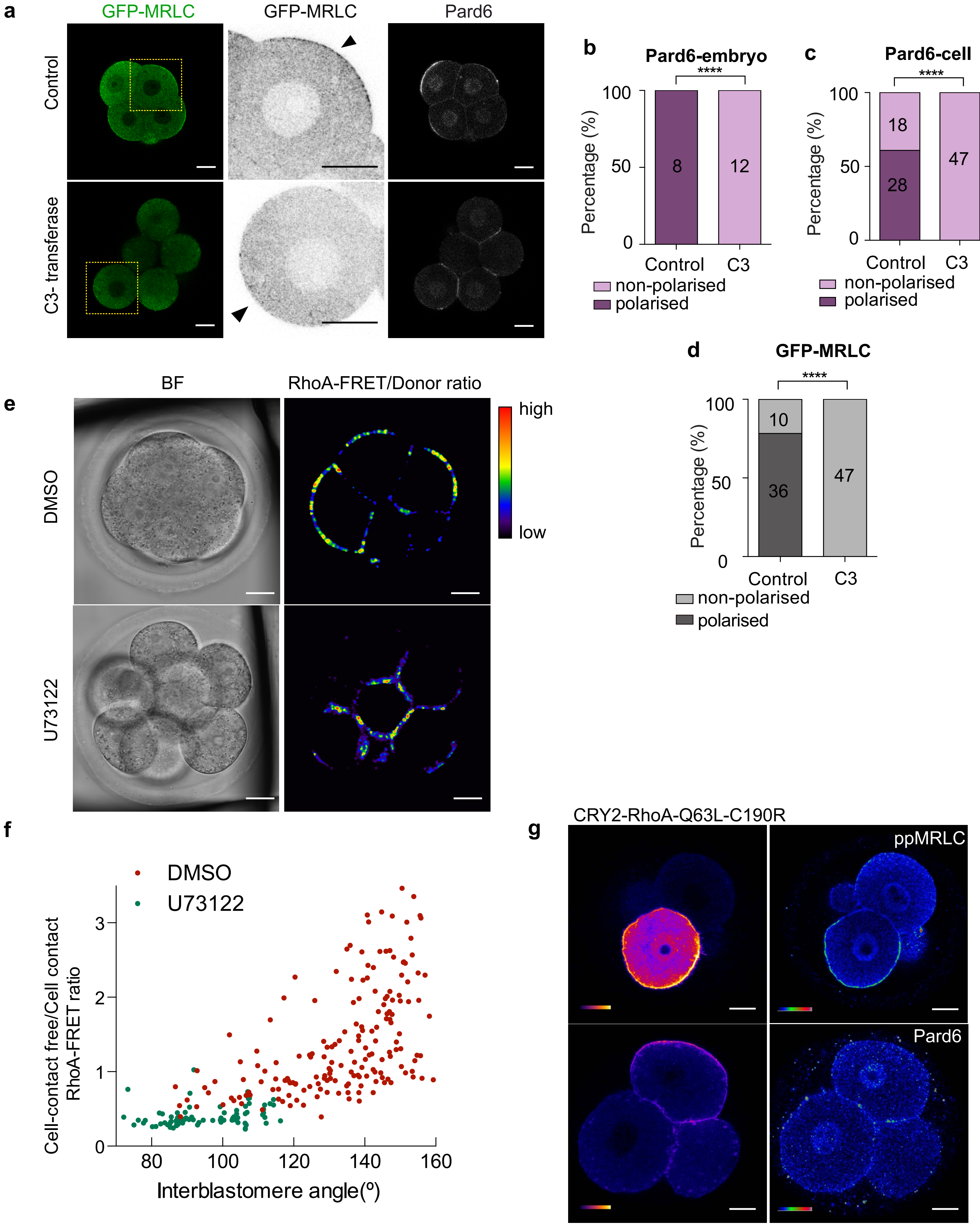

a
GFP-MRLC
GFP-MRLC
Pard6
Control
C3- transferase
b
Pard6-embryo
****
100
50
0
Percentage (%)
8
12
Control
C3
non-polarised
polarised
c
Pard6-cell
****
100
50
0
Percentage (%)
18
28
47
Control
C3
non-polarised
polarised
d
GFP-MRLC
****
100
50
0
Percentage (%)
10
36
47
Control
C3
non-polarised
polarised
e
BF
RhoA-FRET/Donor ratio
high
low
DMSO
U73122
f
DMSO
U73122
Cell-contact free/Cell contact RhoA-FRET ratio
3
2
1
0
80
100
120
140
160
Interblastomere angle(º)
g
CRY2-RhoA-Q63L-C190R
ppMRLC
Pard6

Supplementary Figure 7. C3-transferase treatment abolishes actomyosin and Par complex polarisation.

(**a**) GFP-MRLC overexpressing embryos were treated with C3-transferase and immunostained for GFP and Pard6 (**b-d**) Quantification of polarisation in embryos from panel a. Percentage of embryos showing polarised Pard6 localisation (b), percentage of blastomeres showing polarised Pard6 localisation (c) and percentage of blastomeres showing polarised GFP-MRLC localisation. C3: C3-transferase. The number of embryos per category is indicated in the bars. Data is shown as a contingency table. ****$p<0.0001$, Fisher's exact test. (2 independent experiments). (**e**) RhoA activity in DMSO (control group) and U73122 treated embryos at the late 8-cell stage, visualized using a RhoA FRET sensor (**f**) Cell-contact free/cell-contact RhoA-FRET ratio as a function of interblastomere angle in embryos from panel e. (N=14-16 embryos, 4 independent experiments). (**g**) RhoA was photo-activated at the cortex of 4-cell stage embryos, which were fixed after photo-activation and immunostainned for ppMRLC and Pard6 (N=13 embryos, 4 independent experiments). All scale bars, 15μm.
