# Supplementary material for: Actomyosin polarisation through PLC-PKC triggers symmetry breaking of the mouse embryo
Source: Nat Commun. 2017 Oct 13;8:921. doi: 10.1038/s41467-017-00977-8 (PMC5640629; doi:10.1038/s41467-017-00977-8)
Supplement: Supplementary file 3 — Description of Additional Supplementary Files [file 41467_2017_977_MOESM3_ESM.pdf]

Description of Additional Supplementary Files

File Name: Supplementary Information
Description: Supplementary Figures and Supplementary Table

File Name: Peer Review File

File Name: Supplementary Movie 1
Description: DMSO-treated embryos compact normally at the 8-cell stage. DMSOtreated embryos were filmed from the 4-8 cell stage to the 8-16 cell stage. Time interval = 25 minutes per frame. Scale bar = 15µm.

File Name: Supplementary Movie 2
Description: U73122 treatment prevents compaction at the 8-cell stage. U73122-treated embryos were filmed from the 4-8 cell stage to the 8-16 cell stage. Embryos failed to compact at the 8-cell stage. Note that U73122 treatment did not affect cytokinesis. Time interval = 25 minutes per frame. Scale bar = 15µm.

File Name: Supplementary Movie 3
Description: Ruby mRNA injected embryo compacts at the 8-cell stage. 2-cell stage embryos were injected with Ruby mRNA (as a control) and filmed from the 4-8 cell stage to the 8-16 cell stage. The red channel shows Ruby protein. A maximum projection is shown. Time interval = 20 minutes per frame. Scale bar = 15µm.

File Name: Supplementary Movie 4
Description: PLC-DN mRNA injected embryo fails to compact at the 8-cell stage. 2-cell stage embryos were injected with PLC-DN mRNA and filmed from the 4-8 cell stage to the 8-16 cell stage. Embryos failed to compact at the 8-cell stage but cytokinesis was not affected. The red channel shows PLC-DN protein. A maximum projection is shown. Time interval = 20 minutes per frame. Scale bar = 15µm.

File Name: Supplementary Movie 5
Description: Local illumination with blue light triggers rapid membrane recruitment of the PKC kinase domain. 2-cell stage embryos were injected with CIB1-Zsgreen-CAAX and CRY2-mCherry-PKC-KD. A defined region in the cell-contact free surface was illuminated with 458nm wavelength. The illuminated blastomere was filmed for 10 minutes to monitor the change of CRY2-mCherry-PKC-KD localisation. Time interval = 20 seconds per frame. Scale bar = 15µm.
